# Finite Element Analysis of Various Osteotomies Used in the Treatment of Developmental Hip Dysplasia in Children

**DOI:** 10.3390/jpm14020189

**Published:** 2024-02-08

**Authors:** Zsuzsanna Incze-Bartha, Sandor Incze-Bartha, Zsuzsánna Simon-Szabó, Andrei Marian Feier, Vlad Vunvulea, Alin Ioan Nechifor-Boila, Ylenia Pastorello, Lorand Denes

**Affiliations:** 1 Department of Anatomy, “George Emil Palade” University of Medicine, Pharmacy, Science and Technology of Targu Mures, 540139 Targu Mures, Romaniaylenia.pastorello@umfst.ro (Y.P.); lorand.denes@umfst.ro (L.D.); 2 Department of Orthopedics and Traumatology, “Fogolyan Kristof” County Hospital Sfantu Gheorghe, 520064 Covasna, Romania; 3 Department of Pathophysiology, “George Emil Palade” University of Medicine, Pharmacy, Science and Technology of Targu Mures, 540139 Targu Mures, Romania; 4 Department of Orthopaedics and Traumatology, “George Emil Palade” University of Medicine, Pharmacy, Science and Technology of Targu Mures, 540142 Targu Mures, Romania

**Keywords:** developmental hip dysplasia, children, finite element, geometric model, hip joint, hip osteotomies, femur osteotomies, pediatric hip joint

## Abstract

Late-discovered developmental hip dysplasia deformities often necessitate complex surgical treatments and meticulous preoperative planning. The selection of osteotomies is contingent upon the patient’s age and the specific structural deformity of the hip. In our anatomical hip model, derived from the data of a 12-year-old patient, we performed virtual osteotomies that are commonly recommended for such cases. We precisely constructed geometric models for various osteotomies, including the Dega, Pemberton, Tönnis, Ganz, Chiari pelvic, and Pauwels femoral osteotomies. We employed Autodesk Inventor for the finite element analysis of the hip joint and the corrective osteotomies. In comparing one-stage osteotomies, we noted that the Dega and Ganz pelvic osteotomies, especially when combined with the Pauwels femoral osteotomy, yielded the most favorable outcomes. These combinations led to enhanced femoral head coverage and reduced intra-articular pressure. Furthermore, we calculated the femoral head-to-acetabulum volume ratio for both the Dega and Pauwels osteotomies. The encouraging results we obtained advocate for the integration of finite element analysis in virtual osteotomies of the pelvis and femur as a preoperative tool in the management of developmental hip dysplasia.

## 1. Introduction

Pelvic osteotomies, whether combined with open reduction or not, as well as femoral osteotomies, are surgical interventions indicated and utilized for hip joint deformities in pediatric orthopedics. The primary objective of these procedures is to enhance femoral head coverage, augment the contact surface, and promote the growth and development of the joint in a normative direction. The selection of a specific procedure is contingent upon the type and degree of deformity, as well as the presence of arthrosis [1].

In recent years, corrective osteotomies employed in the treatment of developmental hip dysplasia have been classified into two principal categories: reconstructive and salvage osteotomies. Reconstructive osteotomies aim to restore normal hip anatomy and are subdivided into three groups: acetabuloplasty with distal mobilization of the acetabular roof (Pemberton, Albee–Lance, Dega, San Diego), redirectional osteotomies involving the displacement of pelvic fragments (Salter, Le Coeur, Hopf, Steel, Sutherland–Greenfield, Kalamchi, Tönnis–Trousseau, PemberSal), and pelvic osteotomies with the reorientation of the acetabulum (Blavier, Wagner, Eppright, Ninomiya, Hsieh, Ganz–Bernese). Salvage osteotomies, recommended post-bone maturity, aim to postpone arthroplasty and provide enhanced coverage of the acetabular component during arthroplasty (Chiari, Wilson, Staheli) [2,3,4,5].

For the correction of coxa valga in hip dysplasia, the most frequently utilized osteotomies are in the intertrochanteric area, specifically the Pauwels varus derotational osteotomies. Depending on the clinical requirements, the femoral osteotomy can be executed at the trochanteric or subtrochanteric level.

Complex surgical procedures that encompass pelvic osteotomies, femoral osteotomies, and open reduction are termed one-stage operations. In the majority of hip dysplasia cases, one-stage surgery is necessitated [6,7,8,9].

The indication for various osteotomies is determined by the patient’s age and the extent of the deformity. In pediatric patients, the presence of growth plates dictates the application of specific types of pelvic and femoral osteotomies. Amidst the numerous indications and techniques for osteotomies in the correction of hip dysplasia, we have moved beyond the traditionally reported literature recommendations that categorize osteotomies based on dysplasia morphology. Instead, we have endeavored to identify the most efficient method through finite element analysis [8].

## 2. Materials and Methods

For the finite element analysis of the hip and corrective osteotomies, we utilized CT images from a 12-year-old female patient. This patient, with normal psychomotor development and a body weight of 32 kg, did not undergo screening for congenital hip dysplasia after birth. At 12 years of age, the patient presented with a painless limping gait, a positive Trendelenburg sign on the left, increased lordosis, a positive Galeazzi sign on the left, and a limitation of left hip abduction to 30 degrees. The pelvic radiographic findings included: an interruption of the Shenton–Menard line, an acetabular index of 35 degrees, and a Wieberg angle of 3 degrees, indicating developmental dysplasia of the left acetabulum with a subluxated valgus femur. The recommended treatment was a surgical one-stage operation involving both pelvis and femur osteotomies. Before surgery, the patient underwent a CT scan of the affected hip.

### 2.1. The Chosen Osteotomies

From the multitude of osteotomies employed in the treatment of developmental hip dysplasia, we have chosen to model the most frequently used ones, specifically focusing on osteotomies at the pelvic and femoral levels.

Prior to the closure of the Y cartilage, when bone tissue remains elastic (ages 2 to 12 years), reformation osteotomies or acetabuloplasties from pelvic reconstructive osteotomies are employed in the treatment of developmental hip dysplasia. These procedures are particularly recommended for acetabular defects in the upper or anterior region. The advantage of acetabuloplasties lies in their ability to restore the normal acetabular angle and cover the femoral head with hyaline cartilage. The most utilized techniques in this context are the Pemberton and Dega osteotomies.

Other types of osteotomies that avoid the growth cartilage but still ensure femoral head coverage are redirectional osteotomies. Redirectional osteotomies are indicated in cases of severe dysplasia where acetabuloplasty is insufficient to achieve desirable outcomes. These osteotomies can be single, double, or triple pelvic procedures. The most used redirectional osteotomies are the Salter and the Tönnis–Trousseau ones.

If the Y cartilage is closed or close to closure, pelvic osteotomies with acetabular reorientation are used to cover the acetabular head. These osteotomies involve releasing the acetabulum along a curved or polygonal osteotomy line and rotating it in the desired direction. Approximately 20 techniques are described in this context. In this group, we find the technically challenging Ganz osteotomy, which is nowadays the most frequently used.

In adult patients with slightly painful hips due to developmental dysplasia, salvage-type pelvic osteotomies can offer periodic pain relief. These salvage osteotomies also create a stable bony surface for the acetabular component of total hip arthroplasty. The described techniques differ from each other. In the Wilson and Staheli technique, a bony shelf is formed above the femoral head. One utilizes the outer acetabular roof cortex to create a lateral support for the femoral head, while the other, under the direct tendon of the rectus femoris muscle, forms an anterolateral acetabular shelf. On the other hand, the Chiari osteotomy mobilizes the whole distal pelvic fragment to cover the femoral head. We have modeled the Chiari one because, among this type of osteotomy, it is the most frequently used.

The proximal femur can also present axial and derotational deformities in developmental hip dysplasia. Usually, the femoral neck and trochanteric region are in valgus and rotated anteriorly. Femoral osteotomies are employed to correct deformities in the proximal part of the femur. The most frequently used technique is the intertrochanteric varus derotation osteotomy of Pauwels (1976). The virtual operations were executed in accordance with the operative techniques delineated in orthopedic surgical treatises [10,11].

### 2.2. Geometric Model Generation and Editing the Osteotomies

For finite element analysis, the models can either be patient-specific or modeled. In our study, we utilized patient volumetric data obtained from computed tomography (CT) scans conducted preoperatively. The CT data were derived from 28 slices with a resolution of 0.613281 × 0.613281 mm/pixel in the XY plane and a slice thickness of 5 mm, presented in 8-bit format.

The CT slices, stored in DICOM format, were processed using MicroDicom 0.8.8 PACS DICOM and converted to JPEG format. Subsequently, the images were imported into ImageJ (Wayne Rasband, 1997) version 1.54c. The Canny edge detector algorithm was applied to delineate the cortical and trabecular bone structures. The 8-bit images were further simplified into binary form, mapping entry points for surface construction. Using non-uniform rational B-spline (NURBS) curves and a proprietary algorithm, we minimized the distortion by reducing the number of control points.

Our in-house software facilitated the overlay of the established outlines, and with the assistance of Autodesk Inventor, we generated surface areas. The resulting three-dimensional model represented the cortical bone, trabecular bone, cartilage, and the contact nonlinearities of the hip joint as separate entities. In the dysplastic hip model, the articular components retained their original positions imported from the CT scans [12].

In the Pemberton–Zanoli technique (1965), we initiated the pericapsular osteotomy between the anterior superior and inferior iliac spines, subsequently curving it posteriorly towards the Y cartilage. On the three-dimensional model, the fragment was mobilized separately in both the anterior and lateral directions [10,13].

**Figure 1:**
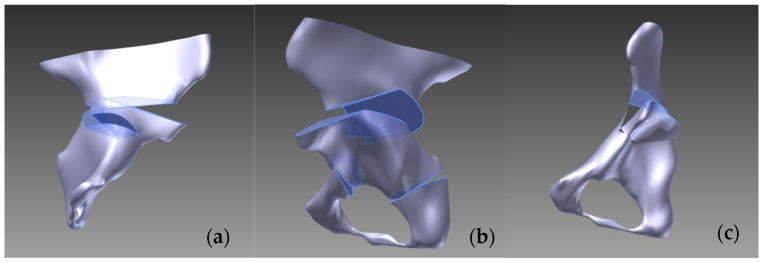
The process of creating (**a**) Pemberton, (**b**) Dega, and (**c**) Tönnis pelvic osteotomies.

In the Dega (1969) pelvic osteotomy model, the osteotomy incision commenced 1.5 cm above the acetabular rim, tracing along the acetabulum over the Y cartilage. Subsequently, the liberated fragment was repositioned anteriorly and laterally. We present the process of creating the Pemberton, Dega, and Tönnis pelvic osteotomies in Figure 1 [5,7,10].

From the redirectional oteotomies, we modeled the Tönnis–Trousseau triple osteotomy (1993), wherein the first osteotomy plane commenced 1.5 cm above the acetabular margin, directed towards the sciatic notch. The second plane was in the ischium, positioned 1 cm from the inferior acetabular margin, and the third plane was in the pubis, 1 cm from the inferior acetabular margin, inclined at 65° on the *x*-axis [14].

Re-orienting pelvic osteotomies involves releasing the acetabulum along a curved or polygonal osteotomy line and rotating it in the desired direction, typically indicated after the closure of the growth cartilage. Approximately 20 techniques are described in this context. The Ganz technique (1988) incorporates five osteotomy planes: the first plane starts under the anterior inferior iliac spine, directed towards the sacroiliac joint, terminating 3 cm short of reaching it. The second plane is situated in the posterior area of the acetabulum, ending before the inferior edge of the pelvis. The third plane is located in the ischium, intersecting with the fourth plane in the inferior extra-acetabular area. The fifth plane is positioned near the transverse cup on the pubis. The mobilized acetabulum is then rotated anterolaterally around the *x* and *z* axes [7,15,16,17].

In the case of the Chiari osteotomy (1950), the osteotomy incision was initiated just above the acetabular margin, taking a slightly oblique direction upwards at a 6° angle towards the sciatic notch. Subsequently, we mobilized the ilium laterally by 1.5 cm [7].

**Figure 2:**
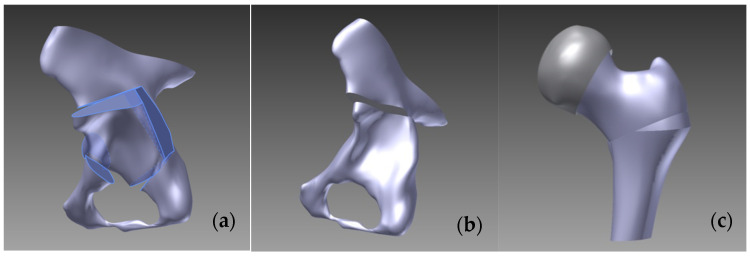
The process of creating (**a**) Ganz, (**b**) Chiari pelvic, and (**c**) Pauwels femoral osteotomies.

For the femoral Pawels osteotomy, we performed a horizontal incision in the intertrochanteric region, after which the separated fragments were repositioned at 120° on the *x*-axis and 15° on the *z*-axis. The process of creating these osteotomies is presented in Figure 2.

In each osteotomy, the osteotomized area was reconstructed using a material identical to bone tissue. Subsequently, we modeled the cartilage in the newly formed acetabulum and femur.

### 2.3. Computational Modell and Overall Assembly

To develop the mathematical model, we assembled the component parts of the model, which, until this stage of the modeling process, were processed separately as individual geometric models. In constructing the geometric model of the normal and displaced pelvis, we were careful not to alter the spatial position of any component, ensuring that both the pelvis and femur remained in their original positions as imported from the CT scans. However, in the hip osteotomy models for the left dysplastic hip, our objective was to maximize the coverage of the femoral head, necessitating modifications to the position of the femur around all axes. As a baseline reference, we performed an “open reduction” of the hip subluxation.

We also executed a rendering where the pelvis was not modified, but the osteotomized (varized) femoral component was positioned in the non-osteotomized dysplastic acetabulum. For the Pemberton osteotomy, the osteotomized femur was displaced 2 mm in the *x*-axis, 1.5 mm in the *y*-axis, and 2 mm in the *z*-axis. In the case of the Dega osteotomy, the displacement was 2 mm in the *x*-axis, 2.5 mm in the *y*-axis, and 2.1 mm in the *z*-axis. For the Tönnis osteotomy, the adjustments were 2.5 mm in the *x*-axis, 3 mm in the *y*-axis, and 2.7 mm in the *z*-axis. For the Ganz osteotomy, the femur was displaced by 2.4 mm in the *x*-axis, 2 mm in the *y*-axis, and 2.3 mm in the *z*-axis. Lastly, for the Chiari osteotomy, the osteotomized femur was repositioned by 1.5 mm in the *x*-axis, 1.1 mm in the *y*-axis, and 0.7 mm in the *z*-axis.

### 2.4. Mathematical Model: Mechanical Properties of Tissues, Boundary Conditions, and Hexagonal Mesh

For the mathematical model, we adopted linear elastic and isotropic material properties characteristic of immature bone tissue. In pediatric patients, the properties of the bone material differ from those in adults, with lower values for Young’s modulus, density, yield stress, and strain. The values for immature bone material properties were sourced from the literature and considered to be an average representation. For the model validation, we utilized adult bone material properties, which were also adopted from the literature [18,19,20,21].

**Figure 3:**
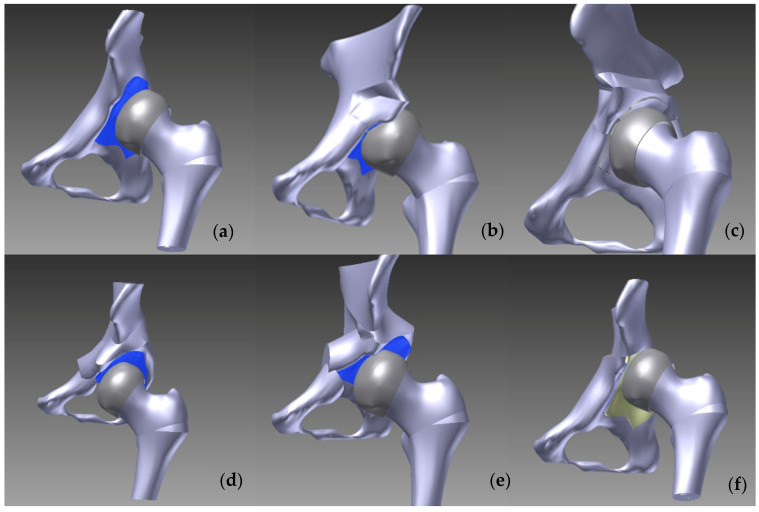
Overall assembly of one-stage operations: (**a**) Pauwels reduction, (**b**) Pemberton–Pauwels, (**c**) Dega–Pauwels, (**d**) Tönnis–Pauwels, (**e**) Ganz–Pauwels, and (**f**) Chiari–Pauwels osteotomies.

The overall assembly of the one-stage operations for the Pauwels reduction, Pemberton–Pauwels, Dega–Pauwels, Tönnis–Pauwels, Ganz–Pauwels, and Chiari–Pauwels osteotomies is presented in Figure 3.

In our model, we differentiated between cortical and cancellous bone tissues in terms of material properties, as highlighted in Table 1. The interface between these areas was considered a fixed contact, devoid of mobility. The articular surfaces were modeled as nonlinear frictionless contact surfaces. Our study incorporated boundary conditions simulating orthostasis on a single leg. The sacroiliac joints were immobilized in the transverse plane, permitting pelvic movement only in the sagittal plane. The distal end of the femur was fixed in all directions.

A vertically directed force of 320 N was applied to the constructed models, centered on the middle of the force-transmitting body of the hemipelvis. This force magnitude was calculated based on the weight of the patient under examination. The balancing force of the abductor muscles was applied along the trajectory of the gluteus medius muscle [22,23,24].

**Figure 4:**
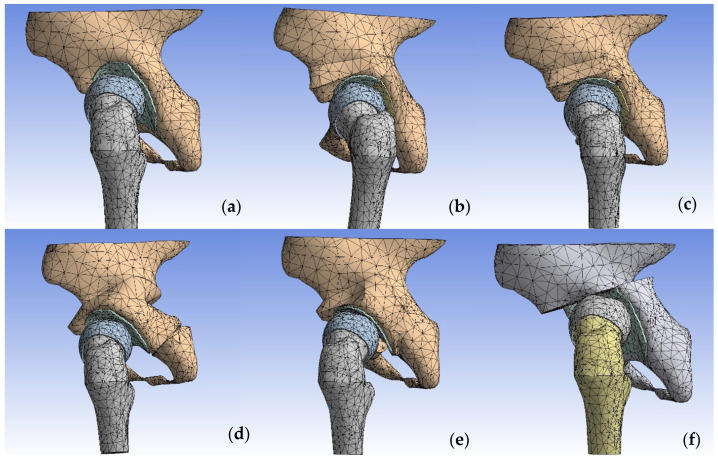
Hexagonal mesh in the: (**a**) left displaced hip with reduced femoral with Pauwels, one-stage operation: (**b**) Pemberton–Pauwels, (**c**) Dega–Pauwels, (**d**) Tönnis–Pauwels, (**e**) Ganz–Pauwels, (**f**) Chiari–Pauwels.

Using Autodesk^®^ Inventor™ 2009 (Autodesk, San Francisco, CA, USA), we generated hexagonal elements on the previously constructed geometric models, as presented in Figure 4. The research conducted by Ramos and Sim demonstrated that the accuracy of finite element models is not significantly dependent on the shape of the finite elements utilized. Hexagonal elements offer greater stability during the analysis process. The quantity of elements incorporated in our constructed model was sufficiently large to ensure a high degree of precision in the pelvic model [25,26].

We meticulously generated each component separately—including the pelvic cortical bone, cancellous bone, and cartilage, as well as the femoral cortical bone, cancellous bone, and cartilage—for each specific model. These models encompassed the non-osteotomized pelvis with Pauwels and reduction, as well as one-stage osteotomies such as Pemberton, Dega, Ganz, Tönnis, and Chiari, combined with the varus-derotation Pauwels osteotomy.

## 3. Results

To comprehend the occurrence of coxarthrosis and the effectiveness of osteotomies, it is imperative to understand the biomechanics of the dysplastic hip, including the distribution of pressure zones and the magnitude of pressure and stress zones in the joint. We utilized the normal hip model as a benchmark for comparison.

The von Mises stress, built based on the invariant of the stress tensor that describes stress in 3D, is useful as it reduces the complex state of stress down to a single number. To obtain a structural analysis of the osteotomized pelvis, we compared the stress distribution across the structure of the normal and operated hips. Our structures were not tested until yield/failure, as we were interested in the response in the stress of the structure to the same load (320 N).

**Figure 5:**
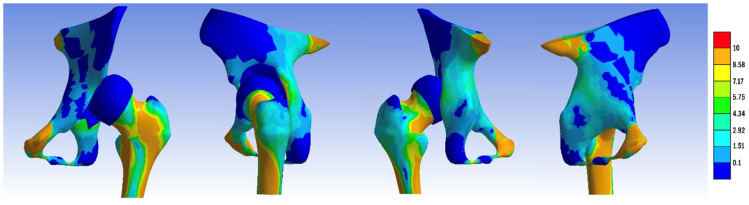
von Mises stress distribution in the untreated pelvis after femoral head reposition.

**Figure 6:**
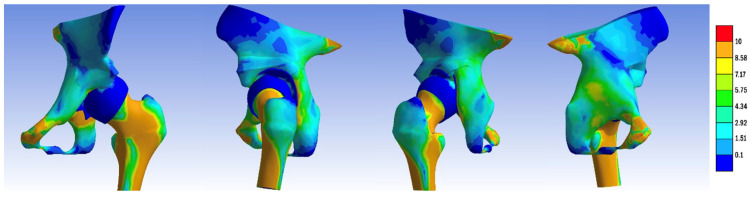
von Mises stress distribution after femoral head reduction, pelvic osteotomy with Pemberton technique, and Pauwels varus-derotational femur osteotomy.

**Figure 7:**
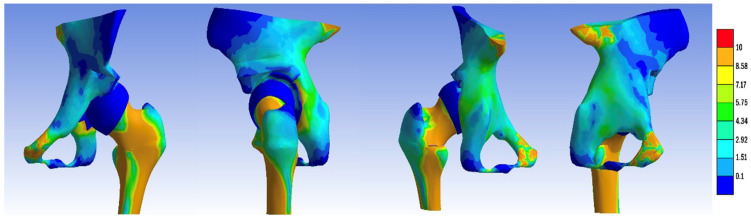
von Mises stress distribution in the treated hip: femoral head reduction, pelvic osteotomy with Dega technique, and Pauwels varus-derotational femur osteotomy.

**Figure 8:**
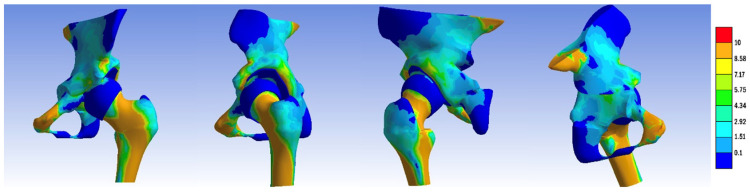
von Mises stress distribution in the treated hip: femoral head reduction, Tönnis pelvic osteotomy, and Pauwels varus-derotational femur osteotomy.

**Figure 9:**
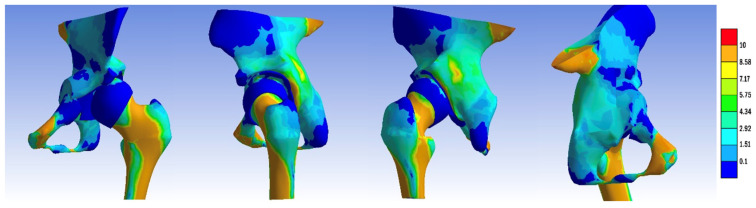
von Mises stress distribution in the treated hip: femoral head reduction, Ganz periacetabular osteotomy, Pauwels varus-derotational femur osteotomy.

**Figure 10:**
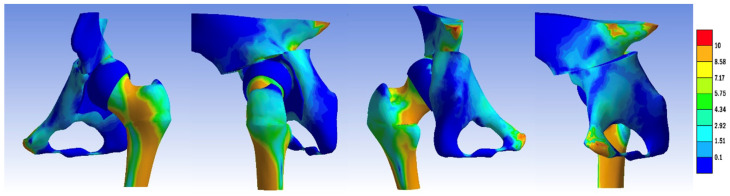
von Mises stress distribution in the treated hip: femoral head reduction, Chiari pelvis osteotomy, Pauwels varus-derotational femur osteotomy.

In the normal hip model, the distribution of the von Mises stress demonstrated a pathway originating from the sacroiliac joint, traversing through the femoral head, and extending into Adams’ arch on the femoral neck, with values ranging from 8.5 to 2.9 MPa. The highest stress values, reaching 8.5 MPa, were observed at the points of constraint of the hemipelvis with the force-transmitting body, the femoral neck, along the trajectory of Adams’ arch, and in the kidney-shaped anterolateral acetabular roof. Corresponding impressions on the femoral head exhibited lower stress values of approximately 2.9 MPa. We present the visual representation of the distribution of the von Mises stress in Figure 5, Figure 6, Figure 7, Figure 8, Figure 9 and Figure 10.

We then compared the von Mises stress zones of other models, including the dysplastic pelvis, open reduction, and one-stage osteotomies (Pemberton, Dega, Ganz, Tönnis, Chiari, Pauwels) with the standard model.

The dysplastic hip model revealed a different stress distribution compared to the normal hip. The stress values in the iliac bone increased in the area of contact with the subluxated femoral head. The stress pathway over Adams’ arch was narrower, while the intertrochanteric area exhibited a broader stress path. Concurrently, the stress zone expanded in size, with values approximating 8.5 MPa.

In all the pelvic osteotomy sites, except for the Chiari technique, there was an increase in the von Mises stress values. The highest stress was recorded at the fourth osteotomy site on the ilium in the Ganz technique (8.5 MPa). In the Dega technique, the pelvic surface of the iliac bone exhibited von Mises stress values of 7.2 MPa. For the Chiari osteotomy, the stress was localized only in the contact area with the femoral head.

The contact surface area was defined as sum of the surface area of the units where the contact pressure was higher than the threshold (0.5 MPa). The calculated maximum pressure was significantly lower than the pressure that would damage the cartilage; however, comparing the results based solely on maximum pressures was not practical.

In the models representing dysplasia and one-stage surgery, we calculated the contact surface area of the acetabulum and compared these values with those observed in the normal hip. During the quantitative comparison of the contact area size, the values that most closely approximated those of the normal hip were observed in the cases of one-stage surgery, specifically the Dega, Tönnis, Pemberton, Ganz, and Tönnis techniques, as visible in Table 2.

Upon measuring the acetabular volume, we discovered that the femur/acetabulum volume ratio decreased following the Dega varus-reduction femur osteotomy. Conversely, this ratio increased in the cases of the Pemberton and Chiari varus-reduction femur osteotomies, and the facts are represented in Table 3.

**Figure 11:**
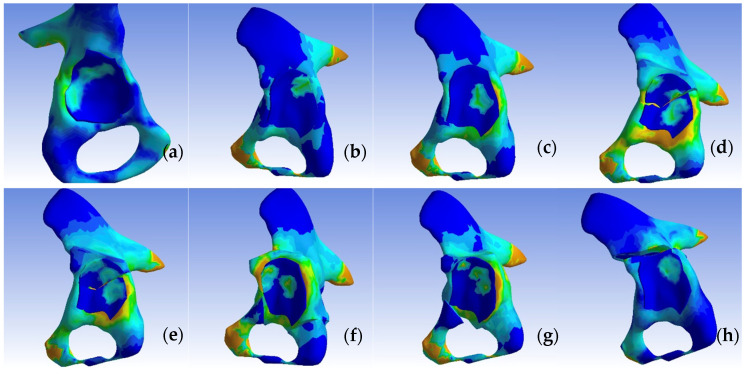
Contact pressure distribution in the acetabulum, non-visualized femoral component: (**a**) normal, (**b**) dysplastic, (**c**) open repositioning, (**d**) repositioning + Pemberton + Pauwels osteotomy, (**e**) repositioning + Dega + Pauwels osteotomy, (**f**) repositioning + Tönnis +Pauwels osteotomy, (**g**) repositioning + Ganz +Pauwels osteotomy, and (**h**) repositioning + Chiari + Pauwels osteotomy.

**Figure 12:**
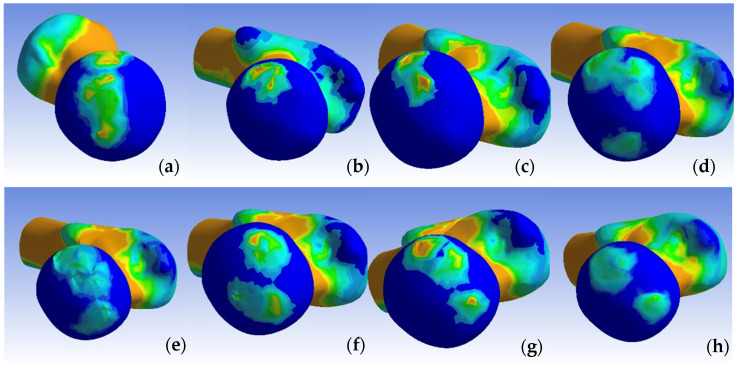
Contact pressure distribution in the femoral head, non-visualized acetabular component: (**a**) normal, (**b**) dysplastic, (**c**) open repositioning, (**d**) repositioning + Pemberton + Pauwels osteotomy, (**e**) repositioning + Dega + Pauwels osteotomy, (**f**) repositioning + Tönnis +Pauwels osteotomy, (**g**) repositioning + Ganz +Pauwels osteotomy, and (**h**) repositioning + Chiari + Pauwels osteotomy.

To enhance the visualization of intra-articular contact pressures, we conducted separate comparisons of the pressure distributions on the femoral head and within the acetabulum across the constructed models, as highlighted in Figure 11 and Figure 12. Notably, in contrast to the dysplasia and Chiari osteotomy models, the stress values decreased, yet the contact zone did not shift towards a normal configuration.

## 4. Discussion

Comprehending the biomechanics of the normal, dysplastic, and osteotomized hip is crucial for the effective treatment of developmental hip dysplasia. Given that in vivo measurements are impractical in pediatric patients, the analysis of the hip joint using the finite element method provides an opportunity for immediate understanding. In clinical practice, it can take months or even years to observe the outcomes from the treatment of dysplasia.

Radiographic evaluation has been widely employed to assess the effectiveness of hip osteotomies for developmental hip dysplasia. The modification in radiological parameters serves as the measure of effectiveness. Numerous studies employing this method have been published, providing insights into the changes in hip morphology during treatment. However, these studies predominantly offer information about the hip architecture in only one or two planes [6,27,28,29].

The advent and increased utilization of computer tomography (CT) have facilitated the three-dimensional visualization of the hip joint. Initially, a significant disadvantage of CT scanning was the requirement for sedation during the examination of children, coupled with the high dose of radiation involved. However, advancements in CT technology have led to enhanced efficiency, resulting in shorter examination times and reduced radiation exposure, thereby rendering this diagnostic tool more practical and safer for use [30].

Over time, this diagnostic tool has become the most widely used paraclinical examination of the congenital dysplastic hip. Tsumura (2005), Klaue (1988), and Tallroth (2006) used CT scans for planning pelvic corrective osteotomies in adults [31,32,33].

With advancements in software and the computer industry, finite element method (FEM) analysis has become increasingly prevalent in orthopedics. This analytical method provides a comprehensive understanding of hip biomechanics, with the added benefit of enabling repeated analyses at any point. Finite element method analysis is particularly effective in quantifying the biomechanical differences between dysplastic and normal hip morphologies.

One of the pioneering studies utilizing computer analysis in this field was conducted by Michaeli et al. in 1998. This study involved a comparison of hip intra-articular pressures calculated through computer analysis with those measured from cadavers. The study concluded that the computerized method accurately replicates both the magnitude and location of intra-articular pressures, thereby validating its utility in planning corrective osteotomies [34].

The majority of studies employing finite element method (FEM) analysis have concentrated on the normal or dysplastic hip in adults. The most thoroughly investigated therapeutic intervention in this context is the implantation of hip endoprostheses. Research on the dysplastic hip has primarily focused on its biomechanics, the validation of mathematical models, and the biomechanical alterations following Ganz, Dial, and Chiari periacetabular osteotomies [24,35,36].

To our knowledge, there are no studies that have examined the effectiveness of various types of corrective osteotomies in treating developmental hip dysplasia in children using finite element method (FEM) analysis. Additionally, there are relatively few FEM studies focusing on the pediatric hip.

Piszczatowski et al. conducted an evaluation of hip biomechanics in children with spastic paraparesis and adductor spasticity using the finite element method. Their study incorporated the increased muscular tone around the hip. The model they used was based on medical images of a 14-year-old patient with spastic paraparesis. The findings of their analysis indicated a medial dislocation of the load-bearing areas in both the femur and acetabulum, attributed to the traction of the adductor muscles [37].

Park et al. developed models using the finite element method based on medical imaging for hips afflicted with femoral head epiphysiolysis and Legg–Calvé–Perthes disease. They executed a shelf pelvic osteotomy on the model for Legg–Calvé–Perthes disease and a femoral osteotomy on the model with femoral head epiphysiolysis. In their finite element analysis of a normal child’s hip, weighing 46.7 kg, the intra-articular pressure was found to be 1.87 MPa. In their models with pelvic osteotomies for Legg–Calvé–Perthes disease, the von Mises stress decreased, and the contact area in the hip joint increased postoperatively. Following the femoral osteotomy for Legg–Calvé–Perthes disease, there was a slight increase in the hip’s contact area, but its distribution remained abnormal. The contact pressure decreased, while the contact force increased marginally [38].

Kim et al. developed finite element models for dysplastic hips using CT data from two patients with dysplastic left hips, one weighing 26 kg and the other 33 kg. They created the solid models using 3D software—Doctor 4.0 for contour detection, and Rapidform software for converting to IGES format. The tetrahedral element mesh necessary for the finite element analysis was generated using the Femap version 8.2 software. The articular cartilage was modeled based on MRI data. They simulated shelf surgery on the dysplastic pelvis in these models. Contact analysis was conducted using the ABAQUS/Standard 6.3 program. Postoperatively, in the first model, the maximum contact pressure decreased from 2.3 MPa to 1.6 MPa, and in the second model, it decreased from 6.0 MPa to 4.5 MPa. The contact area in these models increased by 22% and 23%, respectively [39].

In our model validation analysis, we preserved the geometry of the normal hip and assigned adult bone material properties for the mathematical analysis. We applied a force of 800 N, equivalent to the weight of an adult, to the sacroiliac joint, using the orthostatic position on one leg as a reference. Our results were then compared with in vivo measurements conducted by Bergmann et al. in 2010. Bergmann and colleagues measured forces on the femoral head surface at approximately 2.3 times the body weight. In our model, forces equivalent to 2.5 times the body weight were observed on the femoral head. Comparing our normal adult hip model with the one created by Zhao et al., we noted identical force distributions in both joint components of the normal hip [24].

In our finite element analysis, the von Mises stress distribution on the dysplastic hip model revealed a lateralized path compared to that of the normal hip. In the dysplastic hip, the von Mises stress pathway originated from the sacroiliac joint, directed towards the lesser trochanter through the point of contact of the femoral head with the acetabulum, but with increased stress extending towards the greater trochanter. This path became more medial following pelvic and femoral osteotomies, yet it did not fully align with the position observed in the normal hip.

For our analysis, we employed linear elastic material values characteristic of pediatric bone tissue for each joint component. The von Mises stress values increased after each femoral osteotomy due to the distalization of the femoral head. However, the intra-articular contact area showed significant improvement in cases of reconstructive osteotomies. In the Chiari salvage osteotomy, the contact surface with the posterosuperior area of the acetabulum was maintained, and an additional superior contact area with the iliac bone was established. The intra-articular pressure distribution most closely resembling the normal hip was observed following the Dega and Ganz osteotomies, where the contact area increased by 1.4 and 1.9 times, respectively.

Armiger et al. conducted discrete-element analysis of the dysplastic hip in a group of 12 adult patients with a 10-year postoperative follow-up. They created reconstructions from CT images following a Ganz osteotomy. For their discrete analysis, they utilized triangular elements, applied pressure through the center of the femoral head, and based their calculations on the values of a single elastic element. In their findings, the maximum pressure decreased by an average ratio of 1.7, and the contact area increased by an average ratio of 1.4 [35].

In our analysis, the intra-articular pressures were reduced from 5.7 MPa to values between 3.2 and 4.2 MPa for most procedures, with the exception of the Tönnis osteotomy, where they increased to 7.4 MPa over an area of 50.1 mm^2^.

Through the measurement of the hip joint volume following the periacetabular osteotomies, we established that these types of osteotomies generally do not decrease the acetabular volume. The only exception was observed in the Dega technique, where the femur/acetabulum volume ratio decreased from 0.49 to 0.46 [40].

## 5. Conclusions

In our analysis of virtual treatment for developmental hip dysplasia in a 12-year-old patient weighing 32 kg, the most effective method was identified as the Dega osteotomy combined with femoral head reduction and the Pauwels varus-derotational femur osteotomy. It is plausible that in another patient with a different morphology of hip dysplasia, a different one-stage operation could yield postoperative results closer to normal.

The application of finite element method analysis for examining dysplastic and osteotomized hips offers significant advantages for preoperative planning and preparation. The potential integration of this preparation with intraoperative computer navigation could enable the replication of theoretical results during surgery.

## Figures and Tables

**Table 1:** Bone material property values and boundary conditions used.

Element	Material	Boundary Conditions	Young’s Modulus	Density g/cm^3^	Yield Tensile Strength	Yield Compressive Strength
Cortical bone	Solid	Fixed	11.22 GPa	1.05	35 MPa	62 MPa
Cancellous bone	Solid	Fixed	5.33 MPa	0.6	7 MPa	15 MPa
Cartilage	Solid	Frictionless	0.6 MPa	1.1	1 MPa	1.5 MPa

**Table 2:** Contact area for the various types of osteotomies.

	Contact Area in the Acetabulum (mm^2^)
Normal hip	471.672
Dysplastic hip	205.272
Open reposition	207.576
Pemberton	355.975
Dega	408.619
Ganz	289.123
Tönnis	365.144
Chiari	188.394

**Table 3:** Volumes of the acetabulum, femoral head, and femur/acetabulum ratio in normal osteotomies simulations.

	Acetabulum (mm^3^)	Femur (mm^3^)	Femur/Acetabulum Volume Ratio
Normal	12,095.928	28,360.439	0.42
Dysplastic	8393.275	22,325	0.49
Pemberton	10,942.342	22,325	0.49
Dega	10,302.328	22,325	0.46
Chiari	11,428.190	22,325	0.51

## Data Availability

Data are contained within the article.
